# Ion Mobility QTOF-MS Untargeted Lipidomics of Human Serum Reveals a Metabolic Fingerprint for GNE Myopathy

**DOI:** 10.3390/molecules29215211

**Published:** 2024-11-04

**Authors:** Cristina Manis, Mattia Casula, Andreas Roos, Andreas Hentschel, Matthias Vorgerd, Oksana Pogoryelova, Alexa Derksen, Sally Spendiff, Hanns Lochmüller, Pierluigi Caboni

**Affiliations:** 1 Department of Life and Environmental Sciences, Cittadella Universitaria di Monserrato, Blocco A, Room 13, 09042 Monserrato, Italy; cristina.manis@unica.it (C.M.); m.casula28@studenti.unica.it (M.C.); 2 Department of Pediatric Neurology, Centre for Neuromuscular Disorders, Centre for Translational Neuro- and Behavioral Sciences, University Duisburg-Essen, 45147 Essen, Germany; andreas.roos@uk-essen.de; 3 Children’s Hospital of Eastern Ontario Research Institute, Ottawa, ON K1H 8L1, Canada; aderk098@uottawa.ca (A.D.); sspendiff@cheo.on.ca (S.S.); hlochmuller@toh.ca (H.L.); 4 Department of Neurology, Medical Faculty, University Hospital Düsseldorf, Heinrich Heine University, 40225 Düsseldorf, Germany; 5 Leibniz-Institut für Analytische Wissenschaften, 44139 Dortmund, Germany; andreas.hentschel@isas.de; 6 Department of Neurology, BG-University Hospital Bergmannsheil gGmbH, Ruhr-University Bochum, 44789 Bochum, Germany; matthias.vorgerd@ruhr-uni-bochum.de; 7 Heimer Institute for Muscle Research, BG-University Hospital Bergmannsheil gGmbH, Ruhr-University Bochum, 44789 Bochum, Germany; 8 Directorate of Neurosciences, Royal Victoria Infirmary, Newcastle upon Tyne Hospitals, NHS Foundation Trust, Newcastle upon Tyne NE7 7DN, UK; o.pogoryelova@btinternet.com; 9 Department of Cellular and Molecular Medicine, University of Ottawa, Ottawa, ON K1H 8M5, Canada; 10 Division of Neurology, Department of Medicine, The Ottawa Hospital, Ottawa, ON K1H 8M5, Canada; 11 Brain and Mind Research Institute, University of Ottawa, Ottawa, ON K1H 8M5, Canada

**Keywords:** GNE myopathy, HIBM, Nonaka myopathy, GNEM biomarkers, carnitines, lysophosphocholines, ceramides

## Abstract

GNE myopathy, also known as hereditary inclusion body myopathy (HIBM), is a rare genetic muscle disorder marked by a gradual onset of muscle weakness in young adults. GNE myopathy (GNEM) is caused by bi-allelic variants in the UDP-*N*-acetylglucosamine 2-epimerase (UDP-GlcNAc 2-epimerase)/*N*-acetylmannosamine kinase (ManNAc kinase) gene (*GNE*), clinically resulting in the loss of ambulation within 10–20 years from the onset of the initial symptoms. The disease’s mechanism is poorly understood and non-invasive biomarkers are lacking, hindering effective therapy development. Based on the available evidence, we employed a lipidomic approach to study the serum lipid profile of GNE patients. The multivariate statistical analysis revealed a downregulation of carnitines, as well as of lysophosphatidylcholines, in sera samples derived from GNEM patients. Furthermore, we identified lower levels of sphingomyelins and, concomitantly, high levels of ceramides in serum samples from GNEM patients when compared to control samples derived from healthy donors. Moreover, the GNEM serum samples showed the upregulation of Krebs cycle intermediates, in addition to a decrease in oxaloacetic acid. The correlated data gathered in this study can offer a promising diagnostic panel of complex lipids and polar metabolites that can be used in clinic for GNEM in terms of a metabolic fingerprint measurable in a minimally invasive manner.

## 1. Introduction

*GNEM disease.* GNE myopathy (GNEM), also known as hereditary inclusion body myopathy (HIBM), is a rare genetic muscle disorder marked by a gradual onset of muscle weakness [1]. Nonaka reported for the first time in 1981 three cases of muscle weakness noting a predilection of the phenomenon for the distal extremities [2]. The disease appeared to be inherited in an autosomal recessive pattern. GNEM is caused by bi-allelic variants in the UDP-GlcNAc 2-epimerase/ManNAc kinase gene (GNE) [3]. The GNE gene encodes an enzyme pivotal in the synthesis of *N*-acetylneuraminic acid (NeuAc), which, in turn, binds to glycoproteins and glycolipids, contributing to several functions, including cellular recognition and adhesion. In the initial stage of NeuAc synthesis, UDP-*N*-acetylglucosamine (UDP-GlcNAc) undergoes conversion into *N*-acetylmannosamine (ManNAc) and UDP, facilitated by UDP-GlcNAc-2-epimerase.

The activity of this enzyme is regulated through feedback inhibition by the activated Cytidine-5-monophospho-*N*-acetylneuraminic acid (CMP-Neu5Ac), which serves as the donor of sialic acid [4]. Additionally, CMP-Neu5Ac contributes NeuAc for incorporation into glycoconjugates within the trans-Golgi, facilitated by a family of Golgi-localized enzymes known as sialyltransferases (STs) [5]. However, glycoconjugates can undergo a degradation process in lysosomes, with one of the initial steps involving the removal of a NeuAc residue by neuraminidase, as documented by Sommar and Ellis in 1972 [4]. Finally, the resulting free NeuAc is transported out of lysosomes through a membrane carrier [6].

Several case reports and small cohort studies have depicted the clinical course including the progression of GNE myopathy. Typically, GNEM manifests in the third decade of life, although instances of earlier or later onset have been documented [7]. The disease manifests as a distal myopathy affecting the lower extremities, progressing proximally while normally sparing the quadriceps muscle of the thigh. The involvement of the upper limbs parallels the advancement of the proximal leg muscles.

Muscle biopsies show the presence of rimmed vacuoles that contain several concentric lamellar bodies in various shapes (myeloid and cabbage bodies). Muscle fibers may be necrotic with phagocytosis, but they also lack regenerative processes [2].

Diagnosis and therapy: Diagnosis is usually based on the type of muscle weakness, a modest increase in serum creatine kinase (CK) levels, and occasionally a muscle biopsy showing typical rimmed vacuoles and tubulofilamentous inclusions without evidence of inflammation [8] and is ultimately confirmed by genetic testing. Clinical observations indicate a consistently slow progression of the disease, resulting in the loss of ambulation within 10–20 years from the onset of the initial clinical symptoms [9]. Nevertheless, even with the identification of the gene associated with the disease and a variety of pre-clinical studies existing, there is currently no known approved therapy for GNEM. This is primarily because the pathogenic mechanism of the disease still remains incompletely understood, a challenge compounded by factors like the absence of a suitable model for comprehending the ailment and, consequently assessing potential therapeutic interventions [10], as well as the absence of biomarkers to determine clinical development.

Lipids on GNEM: In 2014, Patzel et al. demonstrated a nonspecific accumulation of glycosphingolipids (GSLs) in a GNE fibroblast model [11]. Specifically, GNE myopathy fibroblasts display elevated GSL levels under normal growth conditions. When these cells are cultured under serum-starved conditions, GSL levels increase even further, indicating that nutrient recycling from the extracellular environment is impaired in the absence of sialic acid. Over time, this impairment could affect the sialylation and function of membrane glycans essential to the endosomal–lysosomal pathway, further restricting access to extracellular sialic acid. Furthermore, a skeletal muscle imaging study of the lower extremities in 31 patients with genetically confirmed GNE myopathy revealed a significant decrease in intramyocellular lipids and trimethylamines, indicating altered muscle metabolism in the early stages [12].

Aim of this present study: Based on the available evidence and recognizing the unresolved issues inherent in the pathological presentation of GNEM, our study seeks to obtain additional insights into the mechanisms of myopathy by defining the associated metabolic fingerprints analyzable in a minimally invasive manner in terms of biomarker profiling. We sought to accomplish this goal by employing a lipidomic approach using patients’ serum, specifically applying an untargeted lipidomic method to analyze the serum lipid profiles of GNEM patients.

In particular, this study reports on the molecular differences, in terms of complex lipids, that emerged from the analysis of the serum of 32 GNEM patients when compared with 22 healthy individuals. Moreover, to pinpoint a disease biomarker capable of indicating the extent of myopathy progression, we examined the lipid profile of an individual patient at different time points through an automated MS/MS approach.

## 2. Results

### 2.1. Untargeted Lipid Analysis

**Figure 1:**
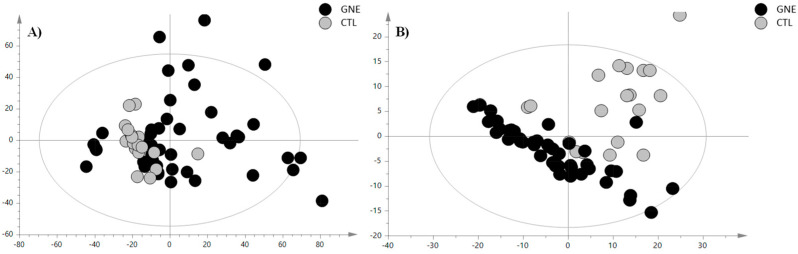
PCA score plot of (**A**) positive ion mode data and (**B**) negative ion mode data. The black circles represent the GNE samples, while the grey circles represent the control samples. The PCA analysis for the PIA showed the following validation parameters: R^2^X = 0.465 and Q^2^ = 0.306; while, for the NIA model, these were R^2^X = 0.654 and Q^2^ = 0.531.

To investigate the distinctive serum lipid profile of GNEM patients and delineate potential variations across different lipid categories compared to healthy volunteers, we used an HPLC-Ion Mobility QTOF-MS platform for sample analysis. The representative total ion chromatograms are depicted in Figure S1. The LC-MS data processing resulted in 465 features for positive ionization analysis (PIA) and 214 features for negative ionization analysis (NIA). These features were subsequently subjected to multivariate statistical analysis (MVA). As a first step, a principal component analysis (PCA) was conducted to examine the sample distribution, identify outliers, and to highlight differences or common features. The unsupervised analysis plots of both the PIA and NIA features are shown in Figure 1.

**Figure 2:**
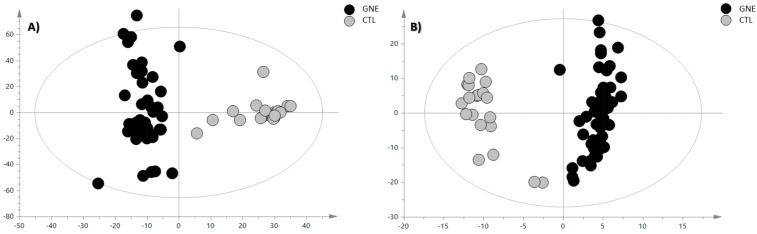
OPLS-DA score plot of (**A**) positive ion mode data and (**B**) negative ion mode data. The black circles represent the GNE samples, while the grey circles represent the control samples. The OPLS-DA analysis for the PIA showed the following validation parameters: R^2^X = 0.336, R^2^Y= 0.906, and Q^2^ = 0.799; while, for the NIA model, they were R^2^X = 0.575, R^2^Y= 0.921, and Q^2^ = 0.865.

For sample classification purposes, we conducted a partial least squares discriminant analysis (PLS-DA) by introducing a dummy Y-variable for each class. An orthogonal modification of PLS-DA (OPLS-DA) was then employed, wherein systematic variations are distinguished into predictive (*x*-axis and interclass variability) and orthogonal (*y*-axis and intraclass variability) components. This approach facilitated the interpretation of group separation variability. The classification and predictive capabilities were assessed using cumulative parameters R^2^Y (goodness of fit) and Q^2^Y (goodness of prediction) determined through the default leave-one-seventh-out cross-validation and tested for overfitting using a y-table permutation test (n = 400) (Figure S2). The OPLS-DA score plots are reported in Figure 2.

The OPLS-DA models demonstrated a robust classificatory power and predictive ability; therefore, the discriminant features were selected based on their VIP values and submitted to the annotation procedure. The discriminant metabolites, annotated through characteristic fragmentation at a collision energy (CE) of 20 eV and through comparison with analytical standards when feasible, are reported in Table 1 and Table 2 for the positive and negative ionization modes, respectively. Additional confidence in these identifications was obtained by comparing the ^DT^CCS_N2_ values of these compounds with those available on the AllCCS database implemented on PubMed, and, when they were not listed there, the Human Metabolome Database (HMDB) database was used. However, when experimentally measured theoretical CCS values were not available, the predicted CCS values provided by these platforms were utilized instead. The error between the theoretical and the measured value of the collision cross section (CCS), indicating how much the measured value deviated from the theoretical value compared to the theoretical value, was calculated as follows:

$$\mathrm{Relative}\;\mathrm{error}\;(\%)=\frac{\left(Measered\;CCS-Theoretical\;CCS\right)}{Theoretical\;CCS}\;\times \;100$$

### 2.2. Iterative MS/MS Analysis

**Figure 3:**
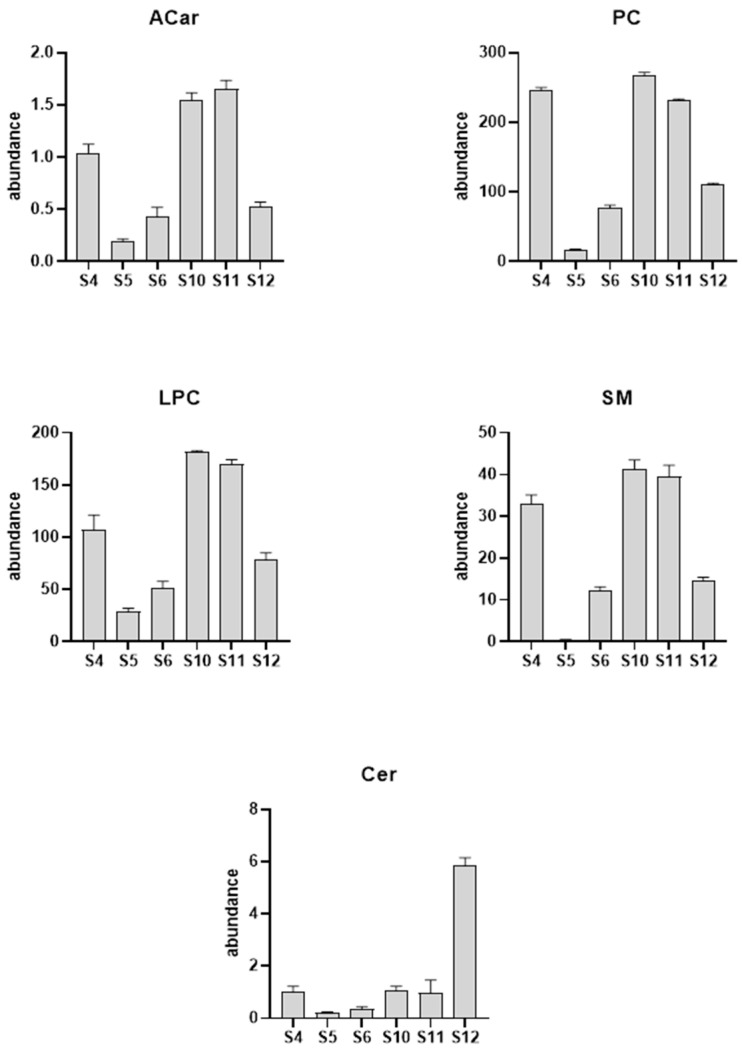
Column plot of the major lipid classes altered due to the GNEM progression. The serum samples used in the longitudinal analysis were collected on visits 4 (S4), 5 (S5), 6 (S6), 10 (S10), 11 (S11), and 12 (S12).

Next, to evaluate how myopathy progression affects the serum lipid profile, we examined three patient’s lipid profile longitudinally. Two female patients, aged 36 and 37, and one male patient, aged 52, had six follow-up visits and serum sample collections over a period of approximately 14 months. Each sample was injected six times in iterative mode with the aim being to identify as many metabolites as possible. Subsequently, using the lipid annotator software, we proceeded to the tentative annotation of the detected metabolites. Then, the chemical identification produced by the lipid annotator software was confirmed with the help of databases such as ID browser, lipid maps, and CEU mass mediator [13]. This complex process was performed for both the positive and negative ionization modes. At the end of the elaboration, we obtained the lipid profile of the sample. The data showed significant alterations in the lipid class of carnitines (ACars), lysophosphocholines (LPCs), and phosphocholines (PCs) in the positive ionization mode. As shown in Figure 3, we observed a decrease in the levels of carnitines, as well as lysophosphocholines, at visit 12 compared to the measurements from the sample collected at visit 4, which occurred over a year earlier. However, for the negative ionization mode, we found a significant increase in the ceramides (Cer) and a concomitant reduction in the sphingomieline (SM) levels.

A more detailed analysis of the complex lipids of each class revealed that the most significant LPC was LPC 18:3 with a reduction of 77%, while, in the PC class, we noticed a significant increase in the levels of PC (18:3_18:0) (+561%) corresponding to the phosphocholine that mirrors the most significant LPC within the LPC class. On the other hand, for the negative ionization mode, we found a significant increase (+91%) in the Cer (d18:1_24:0) and a concomitant reduction in the SM (d18:1_24:0) levels (−99%).

### 2.3. GC-MS/MS Targeted Analysis

**Figure 4:**
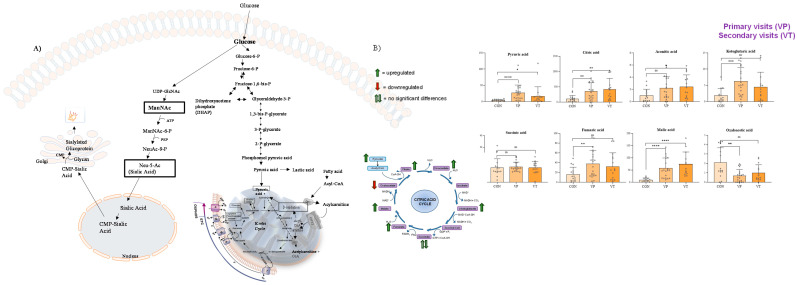
Correlation between sialic acid metabolism and Krebs cycle (**A**) and results of GC-MS/MS analysis of the intermediates of TCA (**B**). In (**B**), VP (or primary visits) indicate the serum collected from visit 1 to visit 5, while VT (or secondary visits) indicate the serum collected from visit 6 to visit 12. Compared to the control samples, GNEM patient-derived samples showed the upregulation of Krebs cycle intermediates, except for succinic acid (which was not found to be significant) and oxaloacetic acid, which was found to be downregulated in GNEM patients. Notably, the most significant altered TCA metabolite was malic acid, which was found to be upregulated in patients showing a *p* value < 0.001, when compared to control samples, at both the first and subsequent visits of the GNEM patients. * *p* value < 0.05; ** *p* value < 0.01; *** *p* value < 0.005; **** *p* value < 0.001; ns *p* value > 0.05.

Given the close correlation between sialic acid metabolism and the Krebs cycle (TCA) in humans (Figure 4A) [14], we decided to perform a targeted analysis of the TCA intermediate metabolites, and the findings are shown in Figure 4B.

## 3. Discussions

### 3.1. Untargeted Lipid Analysis

Currently, diagnosing GNEM relies on clinical muscle pathology findings and confirmation through the identification of biallelic pathogenic variants in the *GNE* gene. Several factors can delay the diagnosis of this rare disease, such as the fact that early-stage clinical manifestations can be nonspecific and the characteristic symptoms, such as quadriceps sparing, do not become evident until the later stages of the disease [14]. In 2013, a nonspecific accumulation of glycosphingolipids in GNEM was reported, suggesting it could serve as a biochemical disease indicator alongside the previously proposed perturbed sialylation state of the neural cell adhesion molecule [11]. However, the accumulation of glycerophospholipids was a common and thus unspecific feature of many other pathological conditions [15,16], leaving the diagnostic challenges for GNEM on a biochemical level partially unresolved, in particular for those cases such as variants of unknown significance (VUSs) or missing cryptic variants (MCVs). To overcome this current limitation, we examined the lipid profile in the serum of GNEM patients in comparison to healthy volunteers using an HPLC-Ion Mobility QTOF-MS analytical platform. The multivariate statistical analysis revealed a downregulation of carnitines in the GNE sera samples (Table 1). The reduction in carnitine levels may indicate a potential change in muscle metabolism along with mitochondrial dysfunction [12]. The notable aspect associated with systemic or muscle carnitine deficiency is a lipid storage myopathy primarily impacting type I muscle fibers, leading to reduced muscle strength and hypotonia [17].

Additionally, the patients with GNEM exhibited low levels of lysophosphatidylcholines (LPCs). After the de novo synthesis of phospholipids, the fatty acyl chains at the sn-2 position are hydrolyzed by phospholipase A2 (PLA2) to produce 1-acyl lysophospholipids. These lysophospholipids then react with lysophospholipid acyltransferase (LPLAT) to incorporate another fatty acid, forming a new phospholipid species [18]. Lysophosphatidylcholine acyltransferases (LPCATs) are specific LPLAT enzymes that regulate the abundance of different LPC species across various cell and tissue types, playing a crucial role in lipid metabolism and homeostasis [19]. In skeletal muscle, LPC is primarily regulated by LPC acyltransferase 3 (LPCAT3), an endoplasmic reticulum membrane protein that preferentially utilizes polyunsaturated fatty acids to acylate LPC [18]. The overexpression of LPCAT3 can reduce the LPC levels in muscles by up to 50%, leading to a significant reduction in skeletal muscle contractile activity, characterized by an approximately 40% loss in force-generating capacity, reduced twitch force, and decreased rates of contraction and relaxation [20]. The reduction in LPC levels observed in GNEM patients may be associated with an overexpression of the enzyme LPCAT3 or a lack of free fatty acids available to be incorporated. Further studies would be needed towards this molecular dissection. Consistently, we found that the serum of GNEM patients presented a downregulation of free fatty acids (Table 2). The low levels of free fatty acids together with the reduced levels of carnitines suggest an alteration in mitochondrial metabolism in the patients affected by GNEM. In this context, it is important to note that the subtle involvement of mitochondrial processes was described in GNEM, representing an unexpected facet of the underlying pathophysiology [21].

Finally, we found lower levels of sphingomyelins and concomitant high levels of ceramides in serum samples from GNEM patients when compared with control samples. This observation is consistent with the mechanism of sphingomyelin synthesis. Indeed, sphingomyelinases (SMases) catalyze the hydrolysis of sphingomyelin to form ceramide and phosphocholine [22]. The regulatory role of ceramides in the pathogenesis of inclusion body myopathy is supported by lipidomic analysis data, which showed a significant increase in ceramides in patients when compared to the control samples [23]. The accumulation of sphingolipid derivatives, such as ceramides, induces cellular toxicity and insulin signaling defects, resulting in insulin resistance [24]. Furthermore, ceramides have the capability to alter the biophysical properties of membranes [25], organizing into microdomains that are rich in cholesterol and sphingolipids. These microdomains facilitate the compartmentalization of receptors and transporters, thereby either promoting or inhibiting various signaling pathways [26]. Hence, in GNEM patients, this aspect might contribute to compromised membrane repair in muscle cells [27].

### 3.2. Iterative MS/MS Analysis

The further evaluation of the serum lipid profile in the context of disease progression, showed a decrease in the levels of carnitines, as well as lysophosphocholine, a significant increase in ceramides, and a concomitant reduction in sphingomyelin levels, confirming the untargeted analysis data. Nevertheless, the iterative MS/MS analysis showed a 91% increase in the specific ceramide (d18:1_24:0) levels in GNEM serum at visit 12 when compared to visit 4. This ceramide is able to inhibit the activation of protein kinase B (PKB) and the translocation of the glucose transporter in skeletal muscle [28]. In addition, elevations in lignoceroyl (C24:0) ceramide in lipoproteins have been demonstrated to raise the levels of the inflammatory cytokines IL-6 and monocyte chemoattractant protein-1 (MCP-1), while reducing the expression of the anti-inflammatory cytokine IL-10 in macrophages [28]. Nevertheless, the significant presence of 24:0 ceramide identified in our study may represent an additional mechanism involved in the development of insulin resistance.

### 3.3. GC-MS/MS Analysis

Active muscle involvement in GNEM is marked by the presence of rimmed vacuoles corresponding to autophagosomes [29], amyloid deposits [30], mitochondrial dysfunction [21,31], and a cellular stress burden [32].

GNE mutant cells exhibited mitochondrial structural changes and alterations in transmembrane potential, indicating a mitochondrial dysfunction [31]. Along this line, in our study, in the GNEM serum samples we identified the upregulation of Krebs cycle intermediates, except for succinic acid, which was not found to be significant, and oxaloacetic acid, which was found to be downregulated in GNEM patients.

The Krebs cycle, catalyzed by seven enzymes in the mitochondria, works in conjunction with the respiratory chain to oxidize nutrients to CO_2_ and produce energy. These specific enzymes of the Krebs cycle can be affected by deficiencies or defects leading to pathological conditions with or without the involvement of the muscular system [33]. Our results showed a significant upregulation of malic acid and a concomitant downregulation of its subsequent intermediate, oxaloacetic acid, suggesting a potential inhibition of the enzyme malate dehydrogenase. A deficiency of malate dehydrogenase is linked to muscular system pathologies as reductions in both isoforms (MDH1 and MDH2) of malate dehydrogenase have been demonstrated in Duchenne muscular dystrophy [34]. To further unveil this pathophysiological interplay, future experiments aimed at the quantitative measuring of these enzymes are needed.

## 4. Materials and Methods

### 4.1. Chemicals

Analytical LC grade, acetonitrile, 2-propanol, ammonium acetate, and formiate, as well as pyridine, methoxamine hydrochloride, and *N*-methyl-*N-*(trimethylsilyl) trifluoroacetamide (MSTFA), were purchased from Sigma-Aldrich (Milan, Italy). Bi-distilled water, (<18 MΩ·cm at 25 °C) was obtained with a MilliQ purification system (Millipore, Milan, Italy). A SPLASH^®^ LIPIDOMIX^®^ standard lipid mixture and 2,2,3,3-D4-succinic acid were purchased from Sigma-Aldrich (Milan, Italy).

### 4.2. Patients

Patients with confirmed homozygous or compound heterozygous disease-causing mutations were recruited for the blood sample donation for research purposes to the JWMDRC Biobank (a member of the EuroBiobank). The ethics approval was granted by Newcastle and North Tyneside 1 Research Ethics Committee, reference number 08/HO906/28 + 5. In this study, there were 32 GNEM patients, evenly split with 16 males and 16 females. The patients’ ages at the time of the serum collection ranged from 29 to 58 years, with an average age of 43 years across all the samples. Longitudinal analysis was conducted on two female patients (aged 37 and 38, respectively) and a male patient, aged 53, involving 6 samples collected over the course of approximately 14 months. The demographic data of the patients in table format, including basic information such as age, gender, genotype, and ambulation status, is reported in Table 3.

### 4.3. Sample Preparation

In order to investigate the changes in the chemical composition, 50 µL of human serum samples were extracted following the Folch procedure [35], using 700 µL of a methanol and chloroform mixture (2:1, *v*/*v*) containing 5 mg/L of 2,2,3,3-D4-succinic acid for the aqueous analysis and Splash lipidomix for the lipid fraction used as the internal standard. The samples were vortexed every 15 min up to 1 h, then 350 µL of chloroform and 150 µL of methanol were subsequently added. The obtained solution was next centrifuged at 17,700 rcf for 10 min, and 400 µL of the organic layer and 200 µL of the aqueous layer were transferred into different glass vials and dried under a gentle nitrogen stream. The dried aqueous layer was derivatized first using 50 µL of methoxyamine hydrochloride dissolved in pyridine at 10 mg/mL, homogenized for 20 s, and kept at room temperature for 17 h. Then, 100 µL of *N*-methyl-*N*-(trimethylsilyl)trifluoroacetamide was added, and the samples were vortexed. After 1 h, 600 µL of hexane was homogenized again before the GC-MS analysis.

The dried chloroform phase was reconstituted with 10 μL of a methanol and chloroform mixture (1:1, *v*/*v*) and 75 μL of an isopropanol–acetonitrile–water mixture (2:1:1 *v*/*v*). All the samples thus prepared were injected in the UHPLC-QTOF-MS/MS apparatus and acquired in the negative ionization mode, while, for the positive ionization mode, they were diluted in a ratio 1:10 *v*/*v*. Quality control (QC) samples were prepared taking an aliquot of 10 μL of each sample and thus analyzed to ensure the quality and accuracy of the analytical method.

### 4.4. UHPLC-IM-QTOF-MS/MS Analysis

The chloroform phase was analyzed with a 6560 drift tube ion mobility LC-QTOF-MS coupled with an Agilent 1290 Infinity II LC system (Agilent Technologies, Santa Clara, CA, USA). An aliquot of 5.0 μL from each sample was injected in a Kinetex C18, 1.6 μm, 100 mm × 2.1 mm chromatographic column (Phenomenex, Bologna, Italy). The column was maintained at 50 °C at a flow rate of 0.5 mL/min. The mobile phase for the positive ionization mode consisted of (A) a 10 mM ammonium formate solution in 60% of milliQ water and 40% of acetonitrile and (B) 90% of isopropanol and 10% of acetonitrile (9:1) containing a 10 mM ammonium formate solution. In the positive ionization mode, the chromatographic separation was obtained with the following gradient: initially 60% of A, then a linear decrease to 50% of A in 2.1 min, then at 30% in 10 min. Subsequently, the mobile phase A was again decreased at 1% and stayed at this percentage for 1.9 min, before being brought back to the initial conditions in 1 min. The mobile phase for the chromatographic separation in the negative ionization mode differed only with the use of 10 mM of ammonium acetate instead of ammonium formate.

An Agilent jet stream technology source, which was operated in both positive and negative ion modes, was used with the following parameters: gas temperature, 250 °C; gas flow (nitrogen), 5 L/min; nebulizer gas (nitrogen), 20 psig; sheath gas temperature, 275 °C; sheath gas flow, 12 L/min; capillary voltage, 4000 V for the positive mode and 3000 V for the negative mode; nozzle voltage, 500 V; fragmentor, 400 V; skimmer, 65 V; octopole RF, 750 V; mass range, 50–1700 *m*/*z*; capillary voltage, 3.5 kV; collision energy, 20 eV in the positive and 25 eV in the negative modes; and mass precursor per cycle = 3. High-purity nitrogen (99.999%) was used as a drift gas with a trap fill time and a trap release time of 2000 and 500 µs, respectively. Before the analysis, the instrument was calibrated using an Agilent tuning solution at the mass range of *m*/*z* 50–1700. An Agilent reference mass mix for mass re-calibration was continuously injected during the run schedule.

The Agilent MassHunter (revision B.09.00) LC/MS acquisition software was used for the data acquisition.

Despite the advancements in high-throughput mass spectrometry, identifying the key structural features of lipids, such as constitutional isomers, carbon–carbon double-bond positions, and acyl chain branching, remains challenging. However, ion mobility coupled with time-of-flight mass spectrometry can address this by separating ions in the gas phase based on their size and shape. This technique enables the separation of ion mixtures according to their mobilities through a gas phase, followed by analysis using a mass spectrometer to measure the mass-to-charge (*m*/*z*) ratios. The primary advantage of this analytical approach is that it provides an additional layer of separation beyond what is achieved with LC alone. This technique provides an extra physicochemical property, the collision cross section (CCS, Å), which, when combined with the retention time and *m*/*z* values, enhances the lipid identification process.

### 4.5. Data Analysis

The data acquired with the LC-MS platform were pre-processed with the software Mass Profinder 10.0 (Agilent Technologies, Santa Clara, CA, USA). This software allowed us to perform the time alignment and deconvolution of the signals, yielding a matrix containing all the features present across all the samples. The mass spectrometer features were filtered based on their presence in the QC samples (threshold = 40%), and the remaining features were collected in a data matrix subsequently processed using SIMCA software 15.0 (Umetrics, Umeå, Sweden). First, a principal component analysis (PCA) was carried out with the aim of observing the distribution of the samples and variables in the multivariate space on the basis of their similarity and dissimilarity. This was followed by a partial least squares discriminant analysis (PLS-DA) with its orthogonal extension (OPLS-DA), which was used as the classificatory model to visualize and evaluate the differences between the sample classes.

### 4.6. Iterative MS/MS Approach

To ensure better sensitivity, the experimental data were also acquired employing the auto-MS/MS technique, operating in an iterative mode with a mass error tolerance of 10 ppm and a retention exclusion tolerance of 0.2 min. In this iterative auto-MS/MS method, the sample underwent multiple injections, and the precursors previously designated for the MS/MS fragmentation were sequentially excluded. Each sample was injected six times at 4 μL each time for the positive acquisition mode and 6 μL for the negative acquisition mode. The data thus acquired were processed using the Agilent lipid annotator software (version 1.0 Build 1.0.54.0) (Agilent Technologies, Santa Clara, CA, USA) as part of a comprehensive lipidomic workflow.

### 4.7. GC-MS/MS Analysis

The aqueous phase was analyzed with a gas chromatograph Trace 1300 coupled with a triple quadrupole mass spectrometer TSQ9000 (Thermo Fisher Scientific Inc., Waltham, MA, USA). Each sample was injected in splitless mode, and the separation was performed on a fused silica capillary column (HP-5MS; 30 m × 0.25 mm i.d., film thickness: 0.25 μm; Agilent Technologies Inc., Santa Clara, CA, USA). The front inlet temperature was 200 °C. Helium gas was used as the GC carrier gas, and argon was used as the MS/MS collision gas. The initial temperature program was as follows: 3 min of isothermal heating at 50 °C, which was then increased to 250 at 3 °C/min and held at 250 °C for 25 min. The transfer line and the ion source temperatures were 280 and 180 °C, respectively. The ions were generated at 70 eV with electron ionization and a dwell time at 1.6 scans/s. Fragment ions were generated using the Automated Selected Reaction Monitoring (AutoSRM) mode based on multiple injections of analytical standards to discover the retention time and product ions and to optimize the collision energies (CEs). The transitions of the metabolites monitored are reported in Table S1.

## 5. Conclusions

Since the first description of the disease entity in 1981 through Nonaka’s studies, significant information has been uncovered on GNEM, both in terms of the genetic landscape [36] and pathological mechanisms [37]. Despite these advances in knowledge, many aspects remain unclear, resulting in the absence of a safe and effective pharmacological treatment for GNEM and a reliable pathophysiology of the disease. This study provides a comprehensive analysis of the lipidomic profile alterations of the serum of patients with GNEM using ion mobility QTOF-MS untargeted lipidomics. Our findings highlighted a marked alteration in several lipid classes, particularly in acylcarnitines (Acars), lysophosphatidylcholines (LPCs), ceramides (Cers), and sphingomyelines (SMs), which are crucial for cellular membrane integrity and energy metabolism in muscle cells. These complex lipids also exhibited longitudinal alterations when the lipid profile of the same patient was analyzed over a two-year period. This evidence suggests that the lipids identified in this study are able to distinguish GNEM profiles from healthy profiles and may be used as predictors of disease severity after a longitudinal study with a larger patient cohort.

Furthermore, our results highlighted a significative alteration of Krebs cycle metabolites. The upregulation of malic acid, along with the downregulation of oxaloacetic acid, suggests the potential inhibition of malate dehydrogenase activity, the deficiency of which is associated with pathologies of the muscular system, which aligns with the clinical manifestations of GNEM.

Future research should focus on expanding these findings through a larger GNEM cohort study, as well as other similar disease cohorts, to determine whether these differential metabolites are specific to GNEM. Additionally, investigating the therapeutic potential of targeting these metabolic pathways should be considered.

The integration of metabolomic and lipidomic approaches will be crucial for advancing our molecular understanding of GNEM and uncovering potential new therapeutic targets.

## Figures and Tables

**Table 1:** Discriminant metabolites between GNE patients and healthy controls, as determined by the OPLS-DA model, along with their corresponding mass spectrometry characteristics. These characteristics were obtained from the positive ionization mode in the mass spectrometry.

Lipid	Adduct	*m*/*z exp.*	*m*/*z theor.*	Δ (ppm)	RT (min)	Formula	^DT^CCS_N2_ (Å^2^) Theoretical	^DT^CCS_N2_ (Å^2^) Measured	Deviation of CCS Values (%)	VIP	*p* Value	Regulation in GNE
Acar 16:0	+H^+^	400.3418	400.3421	0.75	0.82	C_23_H_45_NO_4_	213.80	213.87	0.03	1.08	0.03	↓
Acar 18:0	+H^+^	428.3733	428.3734	0.24	1.14	C_25_H_49_NO_4_	220.50	220.47	−0.01	1.31	0.02	↓
Acar 18:1	+H^+^	426.3593	426.3578	3.5	0.99	C_25_H_47_NO_4_	221.90	218.73	−1.43	1.69	0.006	↓
LPC 15:0	+H^+^	482.3293	482.3281	2.49	1.08	C_23_H_48_NO_7_P	226.34	231.18	2.14	2.12	0.001	↓
LPC 16:0	+H^+^	496.3403	496.3398	1.01	1.12	C_24_H_50_NO_7_P	233.30	231.39	−0.81	1.37	0.007	↓
LPC 17:0	+H^+^	510.3573	510.3554	3.71	1.15	C_25_H_52_NO_7_P	236.07	232.52	−1.50	1.14	0.0002	↓
LPC 18:3	+H^+^	518.3235	518.3241	−1.16	0.89	C_26_H_48_NO_7_P	224.1	224.05	−0.02	2.19	0.0006	↓
LPC 18:2	+H^+^	520.3396	520.3398	0.38	0.98	C_26_H_50_NO_7_P	228.4	227.83	−0.25	1.15	0.005	↓
LPC 18:1	+H^+^	522.3552	522.3554	−0.19	1.21	C_26_H_52_NO_7_P	232.92	233.84	0.39	1.02	0.044	↓
LPC 18:0	+H^+^	524.3711	524.3713	0.38	1.25	C_26_H_54_NO_7_P	238.53	235.15	−1.42	1.34	0.004	↓
LPC 20:4	+H^+^	544.3413	544.3398	2.75	1.18	C_28_H_50_NO_7_P	230.97	234.48	1.51	1.14	0.03	↓
LPC 20:0	+H^+^	552.4013	552.4024	−1.99	1.61	C_28_H_58_NO_7_P	248.6	247.00	−0.64	1.15	0.005	↓
PC 16:0_16:1	+H^+^	732.5533	732.5538	−0.68	3.32	C_40_H_78_NO_8_P	286.5	282.31	−1.46	1.33	0.008	↑
PC 16:1_18:2	+H^+^	756.5563	756.5538	3.30	2.18	C_42_H_78_NO_8_P	282.0	282.88	0.31	1.53	0.02	↓
PC 16:0_18:2	+H^+^	758.5673	758.5694	−2.76	3.25	C_42_H_80_NO_8_P	286.5	287.27	0.27	2.23	0.08	↓
PC 16:0_18:0	+H^+^	762.5993	762.6007	−1.83	3.67	C_42_H_84_NO_8_P	290.8	291.38	0.20	1.03	0.03	↑
PC 35:1	+H^+^	774.6033	774.6007	3.36	2.64	C_43_H_84_NO_8_P	289.8	285.8	−1.38	1.03	ns	↓
PC 35:0	+H^+^	776.61953	776.6164	−1.41	3.98	C_43_H_86_NO_8_P	294.3	286.51	−2.65	1.23	ns	↓
PC 18:1_18:2	+H^+^	784.5823	784.5851	−3.57	2.69	C_44_H_82_NO_8_P	288.2	292.3	1.42	1.25	ns	↓
PC 18:3_20:0	+H^+^	812.6163	812.6164	−0.12	4.63	C_46_H_86_NO_8_P	294.5	296.95	0.83	1.04	0.000006	↓
PC 20:2_20:4	+H^+^	834.5983	834.6007	−2.87	3.57	C_48_H_84_NO_8_P	297.6	299.82	0.75	1.15	0.02	↓
SM 32:2	+H^+^	673.5303	673.5279	3.56	3.24	C_37_H_73_N_2_O_6_P	275.00	274.32	−0.25	1.27	ns	↓
SM 18:0_16:0	+H^+^	705.5903	705.5905	−0.28	3.44	C_39_H_81_N_2_O_6_P	288.2	288.72	0.18	1.07	ns	↓
SM 38:1	+H^+^	759.6353	759.6365	−1.58	5.95	C_43_H_87_N_2_O_6_P	296.7	296.13	−0.19	2.81	0.001	↓
SM 40:1	+H^+^	787.6663	787.6688	−3.17	6.02	C_45_H_91_N_2_O_6_P	299.1	302.02	0.98	1.12	0.02	↓
SM 42:2	+H^+^	815.7003	815.7001	0.24	7.41	C_47_H_95_N_2_O_6_P	304.9	303.91	−0.32	1.46	0.004	↓
SM 43:1	+H^+^	829.7153	829.7157	−0.48	7.83	C_48_H_97_N_2_O_6_P	310.7	310.98	0.09	1.09	0.004	↓
TG 14:0_16:1_18:2	+NH_4_^+^	818.7233	818.7232	0.12	12.61	C_51_H_92_O_6_	305.08	306.96	0.38	1.25	ns	↓
TG 14:0_16:1_18:1	+NH_4_^+^	820.7393	820.7389	0.49	13.54	C_51_H_94_O_6_	310.3	310.24	−0.02	1.26	ns	↑
TG 14:0_16:1_18:0	+NH_4_^+^	822.7553	822.7545	0.97	14.09	C_51_H_96_O_6_	311.5	314.41	0.93	1.14	0.01	↑

↑ = upregulated; ↓ = downregulated; ns = not significant.

**Table 2:** Discriminant metabolites between GNEM patients and healthy controls, as determined by the OPLS-DA model, along with their corresponding mass spectrometry characteristics. These characteristics were obtained from the negative ionization mode in the mass spectrometry.

Lipid	Adduct	*m*/*z Experimental*	*m*/*z* *Theoretical*	Δ (ppm)	RT (min)	Formula	^DT^CCS_N2_ (Å^2^) Theoretical	^DT^CCS_N2_ (Å^2^) Measured	Deviation of CCS Values (%)	VIP	*p* Value	Regulation in GNE
FA 14:0	−H^−^	227.2020	227.2017	1.32	1.19	C_14_H_28_O_2_	149.21	147.32	−1.27	1.58	0.003	↓
FA 16:0	−H^−^	255.2341	255.2337	1.56	1.57	C_16_H_32_O_2_	169.9	169.15	−0.44	1.02	0.0004	↓
FA OH 18:0	−H^−^	299.2603	299.2592	−1.67	1.33	C_18_H_36_O_3_	191.8	196.50	2.45	1.55	0.0006	↓
FA 20:4	−H^−^	303.234	303.233	3.29	1.28	C_20_H_32_O_2_	183.66	186.71	1.66	1.56	0.03	↓
DHA	−H^−^	327.2347	327.233	3.05	1.43	C_22_H_32_O_2_	190.5	190.52	0.01	1.59	0.04	↓
Oleyl arachidonate	−H^−^	553.4906	553.4909	−0.53	4.81	C_38_H_66_O_2_	295.15	296.09	0.32	1.27	0.01	↑
LPE 20:0	−H^−^	508.3407	508.3409	−0.39	1.7	C_25_H_52_NO_7_P	227.41	227.39	−0.008	1.12	0.003	↓
LPG 20:1	−H^−^	537.3202	537.3198	0.74	1.89	C_26_H_51_O_9_P	230.61	230.14	−0.20	1.63	0.03	↓
LPC 18:0	+CHO_2_^−^	568.3604	568.3602	0.35	1.55	C_26_H_54_NO_7_P	248.2	241.54	−2.68	1.15	0.003	↓
PA 16:1_17:2	−H^−^	655.4324	655.4344	−3.05	3.25	C_36_H_65_O_8_P	251.3	255.90	1.83	2.00	ns	↑
PA 16:0_21:0	−H^−^	703.5646	703.5647	−0.14	8.24	C_40_H_81_O_7_P	261.2	258.14	−1.14	1.24	ns	↓
PA 18:0_22:2	−H^−^	741.5794	741.5804	−1.341	9.11	C_43_H_83_O_7_P	278.8	278.45	−0.13	1.26	ns	↓
PG 12:0_16:1	+CHO_2_^−^	709.4303	709.4297	0.84	1.26	C_34_H_65_O_10_P	273.21	272.94	−0.10	2.02	0.0003	↑
Cer 18:0_22:0	+CHO_2_^−^	668.6201	668.6198	0.44	8.56	C_40_H_81_NO_3_	255.98	253.98	0	1.27	0.03	↑
GlcCer 18:0_16:0	−H^−^	700.5735	700.5733	0.28	9.74	C_40_H_79_NO_8_	271.04	272.24	0.44	2.02	0.007	↓
PC 16:0_16:0	+CHO_2_^−^	778.5603	778.5604	−0.12	5.31	C_40_H_80_NO_8_P	284.7	284.98	0.1	1.63	0.006	↓
PC 16:0_18:3	+CHO_2_^−^	800.5445	800.5447	−0.24	4.69	C_42_H_78_NO_8_P	284.6	283.16	−0.50	1.12	0.03	↓
PE 18:0_18:1	−H^−^	744.5546	744.5549	−0.40	3.39	C_41_H_80_NO_8_P	272.91	273.42	0.19	1.28	ns	↓
PE 16:0_20:4	−H^−^	722.5135	722.5131	0.55	3.88	C_41_H_74_NO_7_P	261.8	252.11	−3.70	1.03	ns	↑
PE P-18:1_20:4	−H^−^	748.531	748.5287	3.07	3.95	C_43_H_76_NO_7_P	277.29	283.25	2.15	1.09	0.04	↑
PE 18:0_20:4	−H^−^	766.542	766.5392	3.65	4.37	C_43_H_78_NO_8_P	275.9	275.89	−0.003	1.54	0.006	↑
PE P-20:3_20:4	−H^−^	772.531	772.5287	2.97	3.77	C_45_H_76_NO_7_P	291.14	295.31	1.43	1.33	0.02	↑
PE P-20:2_20:4	−H^−^	774.546	774.5443	2.19	4.57	C_45_H_78_NO_7_P	287.9	292.39	1.55	1.03	ns	↑
SM 16:1_16:0	+CHO_2_^−^	719.536	719.5345	2.08	2.47	C_37_H_75_N_2_O_6_P	267.5	266.35	−0.43	1.27	ns	↓
SM 18:0_16:1	+CHO_2_^−^	747.568	747.5658	2.94	3.11	C_39_H_79_N_2_O_6_P	280.9	280.88	−0.007	1.41	ns	↓
SM d36:1	+CHO_2_^−^	775.598	775.5971	1.16	7.27	C_41_H_83_N_2_O_6_P	286.4	286.37	−0.01	1.22	0.003	↓

↑ = upregulated; ↓ = downregulated; ns = not significant.

**Table 3:** Patient demographic information.

Patient ID	Sex	Age at Sample Collection	Visit Number	Genotype	Ambulation Status
HIM003-13N-S1	F	45	1		Ambulant
HIM006-13N-S1	F	48	1		Ambulant
HIM007-13N-S1	M	45	1		Non-Ambulant
HIM008-13N-S1	M	43	1		Non-Ambulant
HIM009-13N-S1	M	54	1		Ambulant
HIM010-13N-S1	M	56	1	c.650A>G:p.Tyr217Cys and c.2179G>A:p.Val727Met	Ambulant
HIM013-14N-S1	F	36	1	c.650A>G:p.Tyr217Cys and c.2179G>A:p.Val727Met	Ambulant
HIM014-14N-S1	F	38	1	c.2179G>A:p.Val727Met and c.1652G>A:p.Cys551Tyr	Non-Ambulant
HIM015-14N-S1	M	58	1		Non-Ambulant
HIM016-14N-S1	F	35	1	c.830G>A:p.Arg277Gln and c.2179G>A:p.Val727Met	Ambulant
HIM017-14N-S1	F	30	1		Ambulant
HIM018-14N-S1	M	36	1		Ambulant
HIM019-14N-S1	F	47	1	c.764C>T:p.Thr255Ile and c.764C>T:p.Thr255Ile	Ambulant-W/C
HIM020-14N-S1	M	35	1	c.764C>T:p.Thr255Ile and c.764C>T:p.Thr255Ile	Ambulant
HIM021-15N-S3	F	41	3	c.1225G>Tp:Asp409Tyr and c.1985C>T:p.Ala662Val	Ambulant
HIM023-15N-S1	M	50	1	c.1985C>T:p.Ala662Val and c.1985C>T:p.Ala662Val	Ambulant
HIM024-15N-S1	M	34	1		Ambulant
HIM025-15N-S1	F	42	1		Non-Ambulant
HIM302-16N-S11	F	48	11		Ambulant
HIM303-16N-S10	M	29	10	c.331G>T:p.Asp111Tyr and c.1853T>C:p.Ile618Thr	Ambulant
HIM304-16N-S11	F	51	11	c.479G>A:p:Arg160Gln and c.922C>T:p:Arg308Trp	Ambulant
HIM305-15N-S2	F	38	2		Ambulant
HIM305-17N-S12	F	39	12		
HIM306-17N-S11	F	37	11		Ambulant
HIM309-15N-S1	M	31	1	c.1664C>T:p.Ala555Val and c.1853T>C:p.Ile618Thr	Ambulant
HIM309-17N-S10	M	32	10		
HIM310-17N-S10	F	39	10	c.2179G>A:p.Val727Met and c.1034C>T:p.Pro345Leu	Ambulant
HIM311-16N-S11	M	53	11	c.841C>A:p.Leu281Met and c.1225G>T:p.Asp409Tyr	Ambulant
HIM312-17N-S13	M	56	13	c.1225G>T:p.Asp409Tyr and c.2179G>A:p.Val727Met	Ambulant
HIM314-16N-S1	M	39	1	c.705G>A:p.Trp235* and c.1096C>T:p.Arg366Trp	Ambulant
HIM315-17N-S10	M	53	10		Ambulant
HIM317-16N-S1	M	51	1	c.1313dupT:p.Ser439Lysfs*6 and c.2179G>A:p.Val727Met	Ambulant
HIM320-16N-S1	F	32	1	c.829C>T:p.Arg277Trp and c.1768G>A:p.Gly590Arg	Ambulant
					Ambulant
HIM306-15N- S4, HIM306-16N-S5,6,10 and HIM306-17N-S11-12	F	35–37	4, 5, 6, 10, 11, 12		Ambulant
HIM307-16N-S4,5,6,10 and HIM307-17N-S11,12	F	37–39	4, 5, 6, 10, 11, 12		Ambulant
HIM311-16N- S4,5,6,10 and HIM311-17N-S11-12	M	52–53	4, 5, 6, 10, 11, 12	c.841C>A:p.Leu281Met and c.1225G>T:p.Asp409Tyr	Ambulant

## Data Availability

Data are contained within the article and Supplementary Materials.

## Supplementary Materials

The following supporting information can be downloaded at https://www.mdpi.com/article/10.3390/molecules29215211/s1, Table S1. Auto Selected Reaction Monitoring transitions monitored in GC-MS-MS analysis. Table S2. A. Raw data of lipid acquired in positive ion mode reporting ion intensity, m/z ratio and retention time; B. Raw data of lipid acquired in negative ion mode reporting ion intensity, m/z ratio and retention time. C. Raw data of lipididomics Iterative MSMS analysis acquired in positive ion mode reporting ion intensity, m/z ratio and retention time. D. Raw data of lipididomics Iterative MSMS analysis acquired in negative ion mode reporting ion intensity, m/z ratio and retention time. E. Raw data of targeted GC MSMS analysis of TCA intermediates. Figure S1. Representative total ion chromatograms of GNE patients’ serum samples (A), compared with samples provided by healthy volunteers and used as control (B). Figure S2. Permutation test (400 permutations) for the PIA model (A) and NIA model (B).

## Abbreviations

Acars	acylcarnitines;
CCS	collisional cross section;
CE	collision energy;
Cers	ceramides;
CMP-Neu5Ac	Cytidine-5-monophospho-*N*-acetylneuraminic acid;
FA	fatty acid;
GlcCer	Glucosylceramide;
GNEM	GNE myopathy;
GSLs	glycosphingolipids;
HIBM	hereditary inclusion body myopathy;
LPCs	lysophosphocholines;
LPCATs	lysophosphatidylcholine acyltransferases;
LPE	lysophosphatidylethanolamine;
LPG	lysophosphatidylglycerol;
LPLAT	lysophospholipid acyltransferase;
ManNac-kinase	*N*-acetylmannosamine kinase;
MCP1	monocyte chemoattractant protein-1;
MCVs	missing cryptic variants;
MDH	malate dehydrogenase;
MSTFA	*N*-methyl-*N*-(trimethylsilyl) trifluoroacetamide;
MVA	multivariate statistical analysis;
NeuAc	*N*-acetylneuraminic acid;
NIA	negative ionization analysis;
OPLS-DA	orthogonal partial least squares analysis;
PA	phosphatidic acid;
PCs	phosphocholines;
PCA	principal component analysis;
PE	phosphatidylethanolamine;
PG	phosphatidylglycerol;
PKB	protein kinase B;
PIA	positive ionization analysis;
PLA2	phospholipase A2;
PLS-DA	partial least squares analysis;
QC	quality control;
SMs	sphingomyelins;
SMases	sphingomyelinases;
TCA	Krebs cycle;
TGs	triacylglycerols;
VUSs	variants of unknown significance;
UDP-GlcNAc	UDP-*N*-acetylglucosamine.
