# Supplementary material for: Ion Mobility QTOF-MS Untargeted Lipidomics of Human Serum Reveals a Metabolic Fingerprint for GNE Myopathy
Source: Molecules. 2024 Nov 4;29(21):5211. doi: 10.3390/molecules29215211 (PMC11547195; doi:10.3390/molecules29215211)
Supplement: Supplementary file 1 [file molecules-29-05211-s001.zip › Supplementary material of.pdf]

Supplementary material of "Ion mobility-QTOF-MS untargeted lipidomics of human GNE serum patients"

Table S1. Auto Selected Reaction Monitoring transitions monitored in GC-MS-MS analysis.

Compound name	RT (min)	Parent ion (m/z)	Product ion (m/z)	Collision Energy (eV)
2,2,3,3-D4-succinic acid (IS)	19.51	251	131	25
2,2,3,3-D4-succinic acid (IS)	19.51	251	119	25
Succinic acid	19.50	247	203	5
Pyruvic acid	15.65	217	131	5
Fumaric acid	19.86	245	133	10
Fumaric acid	19.86	245	171	15
Malic acid	21.83	239	191	5
Oxaloacetic acid	22.60	172	112	10
Oxaloacetic acid	22.60	172	156	5
α-ketoglutaric acid	23.23	318	313	15
α-ketoglutaric acid	23.23	318	128	5
Aconitic acid	24.80	375	211	5
Aconitic acid	24.80	211	183	5
Citric acid	25.65	273	183	10
Citric acid	25.65	273	229	10

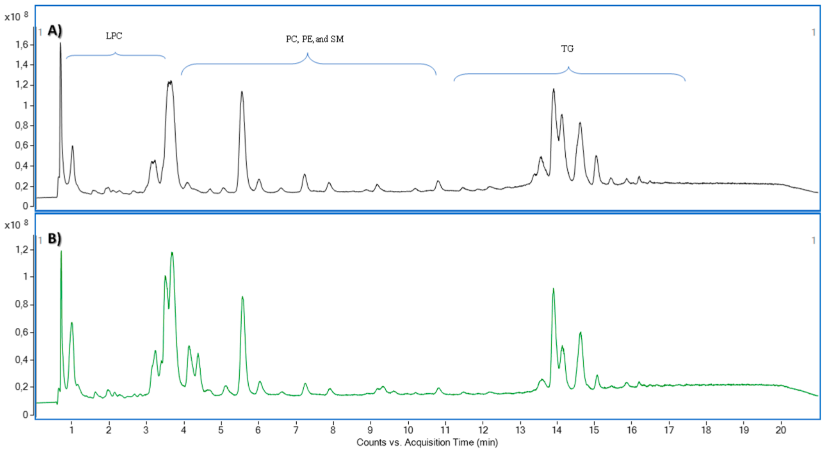

Figure S1. Representative total ion chromatograms of GNE patients' serum samples (A), compared with samples provided by healthy volunteers and used as control (B).

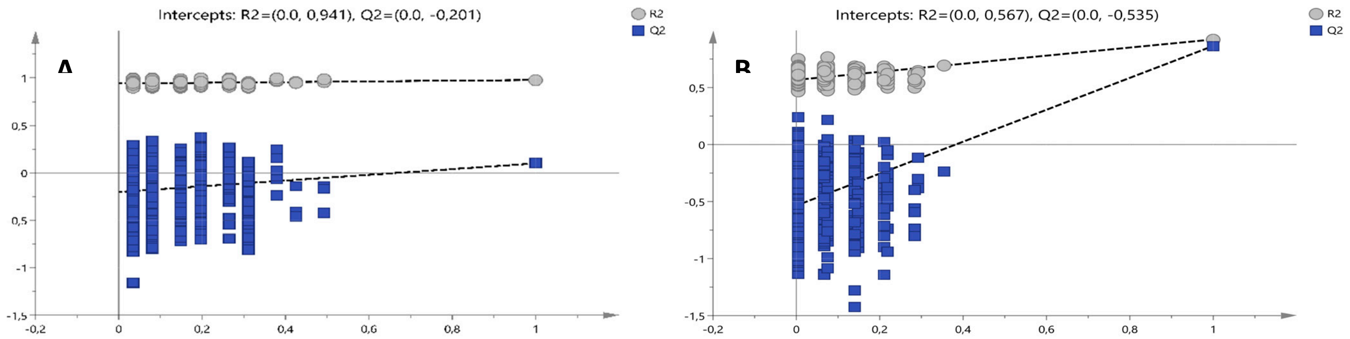

Figure S2. Permutation test (400 permutations) for the PIA model (**A**) and NIA model (**B**).
